# Post-Quantum Secure Lightweight Revocable IBE with Decryption Key Exposure Resistance

**DOI:** 10.3390/e27111160

**Published:** 2025-11-14

**Authors:** Dandan Zhang, Hongwei Ju, Zixuan Yan, Shanqiang Feng, Fengyin Li

**Affiliations:** 1 School of Computer Science, Qufu Normal University, Rizhao 276800, China; zdd2302@qfnu.edu.cn (D.Z.); Yanzixuan@qfnu.edu.cn (Z.Y.); 19547320629@qfnu.edu.cn (S.F.); 2 Experimental Teaching and Equipment Management Center, Qufu Normal University, Rizhao 276800, China; hongweiju@qfnu.edu.cn

**Keywords:** revocable identity-based encryption, decryption key exposure resistance, post-quantum secure, lightweight, lattice

## Abstract

Revocable Identity-Based Encryption (RIBE) can dynamically revoke users whose secret keys have been compromised, ensuring a system’s backward security. An RIBE scheme with decryption key exposure resistance (DKER) guarantees the confidentiality of ciphertext during any time period where the decryption key remains undisclosed. Existing RIBE schemes with DKER generate O(rlog(N/r)) ciphertexts for each plaintext message. Redundant ciphertexts impose significant computational burdens on users and substantial communication overhead on the system. To reduce high computation and communication overhead in existing schemes, this paper proposes a dual-key combination trapdoor generation method. Based on the proposed method, an indirect RIBE scheme with DKER is constructed, reducing ciphertext redundancy and obtaining computation and communication efficiency. Firstly, this paper proposes a dual-key combination trapdoor generation mechanism. By constructing an Inhomogeneous Small Integer Solution (ISIS) instance, the Key Generation Center (KGC) generates and distributes short bases to users as their identity keys. Subsequently, based on the constructed ISIS instance, a new inverse ISIS instance is derived. Furthermore, during each time period, KGC generates short bases for all non-revoked users as their time keys. By linearly combining their identity key with the corresponding time key, every non-revoked user can derive a re-randomized decryption key, achieving controlled key derivation. Secondly, based on the proposed method, a Post-Quantum Secure, Lightweight RIBE scheme with DKER (PQS-LRIBE-DKER) is constructed. For every non-revoked user, their identity key and time key serve as their own user secret key and key update, respectively. Controllable key derivation enables indirect revocation of the scheme. By adopting an indirect revocation, the PQS-LRIBE-DKER scheme achieves a single ciphertext per plaintext message, significantly reducing the sender’s computational load and the system’s communication overhead. Finally, under the hardness assumptions of the Learning with Errors (LWE) and ISIS problems, we prove that the proposed scheme achieves selective identity security in the standard model.

## 1. Introduction

In Identity-Based Encryption (IBE), the users’ identities serve as public keys, obviating digital certificates and eliminating the complex burden of certificate storage and management inherent in traditional public key infrastructures [1]. However, this characteristic also poses a critical challenge for IBE: how to achieve efficient user revocation when faced with key compromise or expiration.

Boneh first proposed a user revocation mechanism for the IBE scheme [2]. In his Revocable IBE (RIBE) scheme, the public key consists of the user identity and time period. The Key Generation Center (KGC) revokes users by regenerating and distributing new secret keys to all non-revoked users at each time period, which incurs a computational overhead proportional to the number of non-revoked users. In practical scenarios, the number of non-revoked users typically far exceeds that of revoked users. Consequently, Boneh’s revocation approach exhibits significant deficiencies in both efficiency and practicality.

To alleviate the efficiency bottleneck, Boldyreva et al. introduced the complete subtree method [3], which avoids generating secret keys individually for every non-revoked user per time period. This scheme assigns users to leaf nodes and calculates the smallest subtree set covering all non-revoked users. KGC generates time keys only for set nodes. This significantly reduces KGC’s computational complexity from O(N−r) to O(rlog(N/r)), where *N *is the total number of users and *r *is the number of revoked users. This scheme achieves revocation by preventing revoked recipients from decrypting messages, which is referred to as indirect revocation.

As research progressed, scholars uncovered new security vulnerabilities in RIBE schemes. In the scheme of [4], the decryption key for each time period is concatenated from the secret key and the key update for that period. Leaking any single period’s decryption key reveals the user’s long-term secret key, thereby compromising ciphertext confidentiality across all time periods. To counter this threat, Seo et al. introduced the decryption key exposure resistance (DKER) and constructed an RIBE scheme with DKER, which guarantees that even after a decryption key compromise, ciphertext confidentiality for all other periods remains secure [5].

Subsequently, RIBE schemes with DKER based on bilinear or multilinear pairings rapidly evolved. However, the rapid advancement of quantum computing threatens to break cryptographic schemes based on traditional mathematical hard problems. Consequently, research on post-quantum secure RIBE schemes with DKER has become an important topic.

### 1.1. Motivations

**Figure 1:**
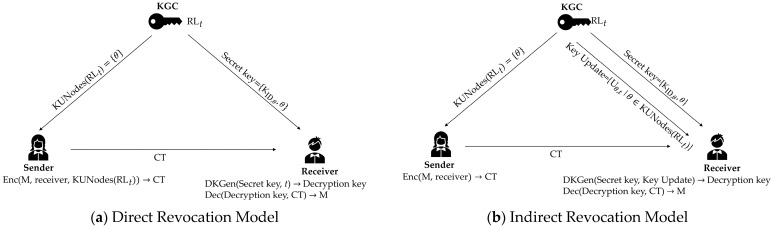
Revocation Model.

To implement DKER, users in Reference [6] autonomously generate re-randomized decryption keys for arbitrary time periods based on their secret keys. As shown in Figure 1a, KGC distributes a short basis corresponding to each user’s identity-based public key matrix as the user’s secret key. Then, the user employs the short basis trapdoor to solve for the randomized short vector corresponding to an identity–time matrix, which serves as the decryption key. This scheme excludes revoked users from the sender’s encryption list to revoke users. The sender has to generate O(rlog(N/r)) ciphertexts for each plaintext message, significantly increasing the computational overhead on the sender and the communication overhead on the system.

In addition to the direct revocation discussed above, IBE also incorporates indirect revocation. As shown in Figure 1b, KGC distributes an identity-related secret key to each user. In each time period, the KGC distributes key updates to non-revoked users. Non-revoked users utilize these two keys to generate their decryption keys. Indirect revocation works by ensuring that revoked users cannot obtain decryption keys, thus losing decryption capability. The sender encrypts without excluding revoked users, effectively reducing the number of ciphertexts required and alleviating the system’s communication overhead. Consequently, designing RIBE schemes that support both indirect revocation and DKER has emerged as a significant research direction.

### 1.2. Contributions

As a solution to the heavy computational burden and high system communication overhead caused by direct revocation in previous RIBE schemes with DKER, this paper proposes a dual-key combination trapdoor generation mechanism to achieve indirect revocation. This enables the construction of a lightweight RIBE scheme with DKER. The contributions are as follows:

- Proposing a dual-key combination trapdoor generation mechanism
  - Starting from an Inhomogeneous Small Integer Solution (ISIS) instance, KGC generates and distributes short bases to users of different identities as their identity keys.
  - In each time period, the KGC constructs a new ISIS instance using the additive inverse of the target matrix and generates short bases as time keys for non-revoked users. Due to the target matrix in the ISIS instance, users cannot directly compute the decryption key for any time period using the short basis alone.
  - Non-revoked users must linearly combine their identity key and time key to obtain the short basis trapdoor corresponding to the public key matrix, thus solving for the current time period’s re-randomized decryption key. By allowing non-revoked users to compute the current decryption key from both keys while denying this capability to revoked users, the scheme successfully achieves controlled key derivation.
- Constructing a post-quantum secure lightweight RIBE scheme with DKER
  - By adopting the above mechanism for key generation and distribution, treating identity keys as user secret keys and time keys as key updates, we construct a Post-Quantum Secure, Lightweight RIBE scheme with DKER (PQS-LRIBE-DKER).
  - Controllable key derivation enables indirect revocation in the scheme. Ultimately, the scheme shifts the periodic computational burden of revocation to a more powerful KGC, maintains a one-to-one correspondence between plaintext and ciphertext, and significantly reduces the sender’s computational load and the system’s communication overhead.
- Providing rigorous formal security proofs
  Assuming the hardness of the Learning with Errors (LWE) and ISIS problems, the proposed scheme has been proven to possess selective identity security in the standard model.

### 1.3. Related Works

Boneh et al. proposed the first RIBE scheme, which relies on traditional bilinear pairing assumptions [2]. In 2012, building upon the work of Agrawal et al. [7] and incorporating the complete subtree method [3], Chen et al. presented the first lattice-based RIBE scheme [8]. In 2016, Nguyen et al. proposed the first lattice-based server-aided revocable IBE scheme [9], which significantly reduces the users’ computational burden through an innovative server-assisted revocation mechanism. In 2021, Zhang et al. presented the first lattice-based RIBE with server-aided ciphertext evolution, preventing revoked users from accessing ciphertexts created prior to revocation [10]. In 2022, Zhang et al. introduced a scalable revocable IBE scheme with cloud-aided ciphertext update, achieving constant-size ciphertexts and a streamlined update mechanism [11]. In 2023, Chen et al. proposed a more efficient cloud-assisted ciphertext-evolving RIBE scheme [12]. In 2024, Huang et al. proposed a generic framework for post-quantum secure identity-based matchmaking encryption with revocable decryption keys, constructed from an identity-based signature scheme and a revocable hierarchical IBE scheme [13]. In 2025, Takayasu et al. presented an RIBE scheme based on the Middle-Product LWE (MPLWE) hardness assumption, achieving the shortest master public key and ciphertext lengths [14].

In 2013, Seo and Emura first introduced the concept of DKER and constructed the first RIBE scheme with DKER based on bilinear pairings [5]. In 2017, Takayasu and Watanabe proposed a lattice-based RIBE scheme satisfying Bounded-DKER (B-DKER), a security model weaker than full DKER [15]. In 2019, Katsumata et al. realized a hierarchical RIBE scheme with DKER and proved its security against selective-identity attacks in the standard model [16]. In 2023, Wang et al. introduced an Enhanced DKER (En-DKER) security notion and constructed a lattice-based RIBE scheme with En-DKER supporting direct revocation [6]. In 2025, Huang et al. proposed an integrated revocation model and used it to construct an efficient lattice-based online/offline integrated revocable IBE scheme with En-DKER [17].

In terms of applications, in 2021, Xia et al. employed an adaptively chosen-ciphertext secure RIBE scheme based on bilinear pairings for IoT device authentication [18]. RIBE schemes have also demonstrated significant potential in authenticated key exchange and proxy re-encryption [19,20]. In 2025, Zhu et al. proposed a revocable hierarchical identity-based inner-product functional encryption scheme with a malicious user revocation mechanism, designed to address patient privacy concerns in smart healthcare systems [21].

## 2. Preliminaries

This paper assumes line vectors. Bold uppercase letters denote matrices, e.g., ***A***. Bold lowercase letters denote vectors, e.g., ***b***. R represents the set of real numbers, Z represents the set of integers, and N represents the set of natural numbers. $\left|\left|x\right|\right|\;$ denotes the $l_{2}$ norm of vector ***x***. The norm of a matrix is the largest $l_{2}$ norm among its rows: $\left|\left|X\right|\right|={max}_{i}||x_{i}||$.

**Definition 1.** **(Full-Rank Differences).** *Let q be a prime and n be a positive integer. We say that a function H:* $Z_{q}^{n}\to Z_{q}^{n\times n}$ *is an encoding with full-rank differences if the following criteria are met:*

1. *For all distinct **u**, **v*** $\in Z_{q}^{n}$ *, the matrix* $H\left(u\right)-H(v)\in Z_{q}^{n\times n}$ *is full rank;*
2.
H *is computable in polynomial time in nlogq.*

### 2.1. Lattice and Discrete Gaussians

Let $B=\{b_{1},\ldots ,b_{n}\}\subset R^{n}$ be a set of *n *linearly independent vectors. The *n*-dimensional lattice generated by the basis ***B*** is defined as

$$\Lambda =L\left(B\right)=\left\{Bc=\sum_{i\in \left[n\right]}c_{i}\cdot b_{i}:c\in Z^{n}\right\}.$$

Let *n*, *m*, and *q *be positive integers. For a matrix $A\in Z_{q}^{n\times m}$, this paper employs two types of *m*-dimensional full-rank integer lattices defined by ***A***:

$$\Lambda_{q}^{⊥}\left(A\right)=\left\{e\in Z^{m}:Ae=0\;mod\;q\right\}\;\;\Lambda_{q}^{u}\left(A\right)=\{e\in Z^{m}:Ae=u\;mod\;q\;,\;u\in Z_{q}^{n}\}$$

For any s>0, the Gaussian function on $R^{n}$ with center ***c*** and parameter *s *is defined as follows:

$$\forall x\in R^{n},\rho_{s,c}\left(x\right)=exp(-\pi {\left|\left|x-c\right|\right|}^{2}/s^{2}).$$

For any $c\in R^{n}$, and real number s>0, an *n*-dimensional lattice Λ, the discrete Gaussian distribution is defined as follows:

$$\forall x\in \Lambda ,D_{\Lambda ,s,c}\left(x\right)=\frac{\rho_{s,c}\left(x\right)}{\rho_{s,c}\left(\Lambda \right)}.$$

For any ordered set of *n *linearly independent vectors $S=\{s_{1},\ldots ,s_{n}\}\subset R^{n}$, let $\tilde{S}=\{\tilde{s_{1}},\ldots ,\tilde{s_{n}}\}$ denote the Gram–Schmidt orthogonalized vectors of ***S***.

### 2.2. Useful Facts

**Lemma** **1.** **(Leftover Hash Lemma [7]).** *Suppose that m > (n + 1)* $\;\log_{2}q+\omega (\log n)$*and that q > 2 is prime. Let **R** be an m × k matrix chosen uniformly in *${\left\{-1,1\right\}}^{m\times k}\;mod\;q\;$*where k = k(n) is polynomial in n. Let **A** and **B** be matrices chosen uniformly in *$Z_{q}^{n\times m}$*and *$Z_{q}^{n\times k}$*respectively. Then, for all vectors **w** in *$Z_{q}^{m}$*, the distribution (**A**, **AR**,*$\;R^{T}w$*) is statistically close to the distribution (**A**, **B**, *$R^{T}w$*).*

**Lemma** **2.** **(Smudging Lemma [22]).** *Let *$B_{1},B_{2}$*be two polynomials over the integers, and let *$D={\left\{D_{\lambda }\right\}}_{\lambda }$*be any *$B_{1}$*-bounded distribution family. Let *$U={\left\{U_{\lambda }\right\}}_{\lambda }$*be the uniform distribution over the integers in [−*$B_{2}\left(\lambda \right),B_{2}\left(\lambda \right)$*]. The family of distributions *D+U*are statistically indistinguishable if there exists a negligible function negl (*·*) such that for all *λ∈N*it holds that *$B_{1}(\lambda )/B_{2}(\lambda )\leq \;$*negl* (λ).

### 2.3. Sampling Algorithms

**Lemma** **3.** **([7]).***Let n* ≥1*,m*$\;\geq 2n\left\lceil \log q\right\rceil$*, q*  ≥*2*

TrapGen (q,n)→(A,S)*: On input q, n, output a pair (*$A\in Z_{q}^{n\times m},S\in Z^{m\times m}$*) such that **A** is statistically close to a uniform matrix in *$Z_{q}^{n\times m}$*and **S** is a basis for *$\Lambda_{q}^{⊥}\left(A\right)$*satisfying *$\left||S||\leq O(\sqrt{n\log q}\right)$*and *$\left||\tilde{S}|\right|$ $\leq O(\sqrt{n\log q})$.
  $SampleLeft\;(A,M_{1},T_{A},u,\sigma )\to e$*: On input a rank n matrix **A** in *$Z_{q}^{n\times m}$*, a matrix *$M_{1}\in Z_{q}^{n\times m_{1}}$*, a short basis *$T_{A}$*of *$\Lambda_{q}^{⊥}\left(A\right)\;and\;a\;vector\;u\in Z_{q}^{n}$*, a Gaussian parameter *$\sigma >||\tilde{T_{A}}||\cdot \omega (\sqrt{\log\left(m+m_{1}\right)})$*, output a vector* $\;e\in Z^{m+m_{1}}$*sampled from a distribution statistically close to *$D_{\Lambda_{q}^{u}\left(\;[A|M_{1}]\right),\sigma }$.
  $SampleRight\;(A,G,R,T_{G},u,\sigma )\to e$*: On input a rank n matrix **A** in *$Z_{q}^{n\times m}$*, the gadget matrix **G** and its trapdoor *$T_{G},\;a\;uniform\;random\;matrix\;R\in {\left\{-1,1\right\}}^{m\times m}$*, and a Gaussian parameter *$\sigma >||\tilde{T_{G}}||\cdot \sqrt{m}\cdot \omega (\sqrt{\log m})$*, output a vector* $\;e\in Z^{2m}$*sampled from a distribution statistically close to *$D_{\Lambda_{q}^{u}\left(\;[A|AR+G]\right),\sigma }$.

### 2.4. Hardness Assumptions

**Definition** **2.** ($Decision-{LWE}_{n,q,\chi ,m}$ [23]). *Given m independent samples *$(a_{i},b_{i})\in Z_{q}^{n}\times Z_{q}$ *where every sample is distributed according to either (1) *$b_{i}=<a_{i}s+e_{i}>$ *with* ${\;e}_{i}\leftarrow \chi$ *for a uniformly random *$s\in Z_{q}^{n}\;$ *(fixed for all samples), or uniform distribution, distinguish which is the case with non-negligible advantage.*

**Definition** **3.** (**ISIS** [24]). *The Inhomogeneous Small Integer Solution problem is as follows: given an integer q, a matrix **A***$\;\in Z_{q}^{n\times m}$*, a syndrome *$u\in Z_{q}^{n}$*, and a real *β*, find an integer vector *$e\in Z^{m}$ *such that *Ae=u mod q *and *${\left||e|\right|}_{2}\leq \beta$.

**Definition** **4. (Preimage Sampleable Function)**. *The function *$f_{A}\left(x\right)=Ax\;mod\;q$*maps inputs from the domain *$D_{n}=\{e\in Z^{m}:{\left||e|\right|}_{2}\leq \delta \sqrt{m}\}$*to the range*$\;R^{n}=Z_{q}^{n}$*. For any *$x\in D_{n}$*, the probability that *$f_{A}\left(x\right)$*output a particular value in *$R^{n}$*is the same as any other value in *$R^{n}$.

### 2.5. Complete Subtree

The PQS-LRIBE scheme adopts the complete subtree method from Reference [3] to reduce the periodic workload of KGC. During the setup phase, KGC constructs a binary tree BT, where user identities are assigned and stored at leaf nodes. A user’s secret key is composed of the keys at each node along the path from the root to that leaf. During the key update phase, KGC uses the algorithm **KUNodes** to compute the minimal node set $Node({RL}_{t})$ covering all non-revoked users. It then generates key updates only for nodes within this set. Non-revoked users derive new decryption keys for the current time period based on valid nodes along their path, while revoked users lose decryption capability due to the absence of a valid key update. Let RL denote the revocation list. Let $Path\left(v\right)$ denote the set of all nodes on the path from the leaf node v to the root node (including both the leaf and the root). Let ρ be a node in the BT, and let$\;\rho_{l}$ and $\rho_{r}$ represent its left and right child nodes, respectively.
$$\begin{array}{l} KUNodes(BT,{RL}_{t})\\ X,NodeUP({RL}_{t})\leftarrow \emptyset \\ \forall \;v_{i}\in RL\\ \;\;\;\;\;\;\;\;add\;Path\left(v_{i}\right)to\;X\\ \forall \rho \in X\\ \;\;\;\;\;\;\;\;if\;\rho_{l}\notin X\;then\;add\;\rho_{l}\;to\;NodeUP\left({RL}_{t}\right)\\ \;\;\;\;\;\;\;\;if\;\rho_{r}\notin X\;then\;add\;\rho_{r}\;to\;NodeUP({RL}_{t})\\ \;If\;NodeUP({RL}_{t})=\emptyset \;then\;add\;root\;to\;NodeUP({RL}_{t})\\ \;\;\;\;\;\;Return\;NodeUP({RL}_{t}) \end{array}$$


## 3. Formal Definitions for RIBE with DKER

### 3.1. Scheme Model for RIBE with DKER

An RIBE scheme consists of the six algorithms (Setup, GenSK, KeyUp, Enc, GenDK, Dec). The syntactic definition of the RIBE does not include an explicit revocation algorithm; the revocation is performed simply by adding the user’s identity to the revocation list RL.

- $Setup(1^{\lambda },N)\to$(PP, MSK): Executed by KGC, this algorithm takes a security parameter λ and the maximum number of users *N *in the system as input, generating the system public parameters PP and the system master secret key MSK as output.

- $GenSK(PP,MSK,ID)\to {SK}_{ID}$: Executed by the KGC, this algorithm takes the system public parameters PP, the master secret key MSK, and a user’s identity ID as input, generating a secret key ${SK}_{ID}$ as the output.

- $KeyUp\left(PP,MSK,{RL}_{t}\right)\to {KU}_{t}$: Executed by KGC, this algorithm takes the system public parameters PP, the master secret key MSK, and the revocation list RL as input, generating the key update ${KU}_{t}$ for time period *t *as output.

- Enc$\left(PP,ID,t,m\right)\to {CT}_{ID,t}$: Executed by the sender, this algorithm takes the system public parameters PP, a user’s identity ID, a plaintext message m, and a time period *t *as input, generating a ciphertext ${CT}_{ID,t}$ as output.

- $GenDK\left(PP,{SK}_{ID},{KU}_{t}\right)\to {DK}_{ID,t}$: Executed by the receiver, this algorithm takes the system public parameters PP, a secret key ${SK}_{ID}$, and a key update ${KU}_{t}\;$as input, generating the decryption key ${DK}_{ID,t}$ for the receiver with identity in time period *t *as output.

- $Dec\left({CT}_{ID,t},{DK}_{ID,t}\right)\to m\;$: Executed by the receiver, this algorithm takes a ciphertext ${CT}_{ID,t}\;$and a decryption key ${DK}_{ID,t}$ as input, generating the plaintext message m as output.

**Correctness**: For all $\;\lambda \in N,N\in N,\left(PP,MSK\right)\leftarrow Setup\left(\lambda ,N\right),m\in M,ID\in ID,t\in T$, and any revocation list RL, the RIBE scheme satisfies correctness if the following probability equation holds:

$$Pr\left(m^{\prime }=m|\begin{array}{c} {SK}_{ID}\leftarrow \mathrm{GenSK}\left(PP,MSK,ID\right)\\ {KU}_{t}\leftarrow \mathrm{KeyUp}\left(PP,MSK,{RL}_{t}\right)\\ {DK}_{ID,t}\leftarrow \mathrm{GenDK}\left(PP,{SK}_{ID},{KU}_{t}\right)\\ {CT}_{ID,t}\leftarrow \mathrm{Enc}\left(PP,ID,t,m\right)\\ m^{\prime }\leftarrow \mathrm{Dec}\left({CT}_{ID,t},{DK}_{ID,t}\right) \end{array}\right)=1-negl(\lambda )$$

### 3.2. Security Model for RIBE with DKER

**Figure 2:**
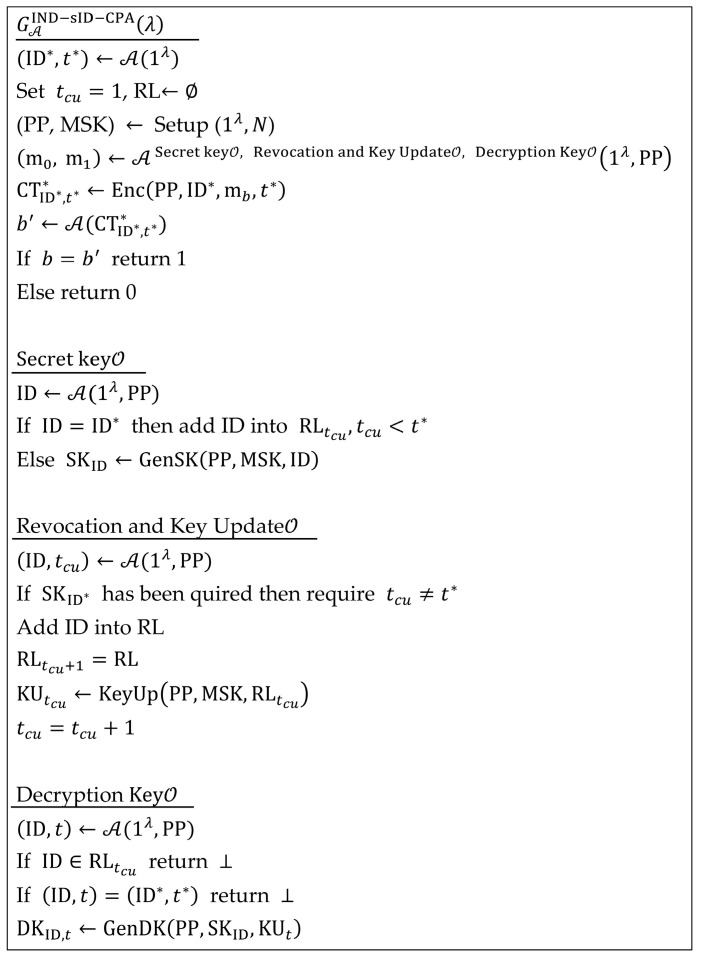
IND-sID-CPA Security Model for RIBE with DKER.

The selective identity security of the RIBE scheme against chosen plaintext attacks is defined through a game between an adversary A and a challenger C in Figure 2. Let the current time period be denoted as $\;t_{cu}$, which is a global variable initialized to 1.

**Definition** **5.** *An RIBE with DKER scheme is selectively secure if the advantage ${Adv}_{A}^{IND-sID-CPA}\left(\lambda \right)=\left|\mathrm{Pr}\left[b=b^{\prime }\right]-\frac{1}{2}\right|$is negligible for any PPT adversaries *A.

To simplify the reduction, this paper categorizes the adversary A into two types:

Type I: A queries the secret key ${SK}_{{ID}^{*}}$ corresponding to the challenge identity ${ID}^{*}.$

Type II: A does not query the secret key ${SK}_{{ID}^{*}}$ corresponding to the challenge identity ${ID}^{*}$.

## 4. Dual-Key Combination Trapdoor Generation Mechanism

KGC directly distributes a short basis trapdoor to users based on their public key matrix, enabling them to compute re-randomized decryption keys for DKER. This allows users to derive decryption keys for any time period using that trapdoor. However, this approach restricts the scheme to direct revocation, resulting in a ciphertext-to-plaintext ratio of $O\left(r\log\left(N/r\right)\right)$, which significantly increases the computational burden on users and the system’s communication overhead. Therefore, designing a more efficient IBE scheme that supports indirect revocation and DKER to enable lightweight user computation has become a critical research focus.

To overcome the limitations of the aforementioned schemes, this chapter proposes a dual-key combination trapdoor generation mechanism. First, KGC generates and allocates a short basis to each user based on an ISIS instance, which serves as their identity key. Subsequently, a new ISIS instance is constructed by taking the additive inverse of the original target matrix. Building upon this derived instance, KGC generates short bases as time keys for non-revoked users across different time periods. Due to the target matrix in the ISIS instance, users cannot directly compute decryption keys using only their identity keys. Non-revoked users must linearly combine their identity key with the corresponding time key. This combination neutralizes the target matrix in the ISIS instance, yielding the short basis trapdoor associated with the public key matrix, which in turn enables the computation of a re-randomized decryption key. Crucially, since time keys are only valid for non-revoked users, revoked users are unable to compute any valid decryption key, thus achieving controlled key derivation.

**Figure 3:**
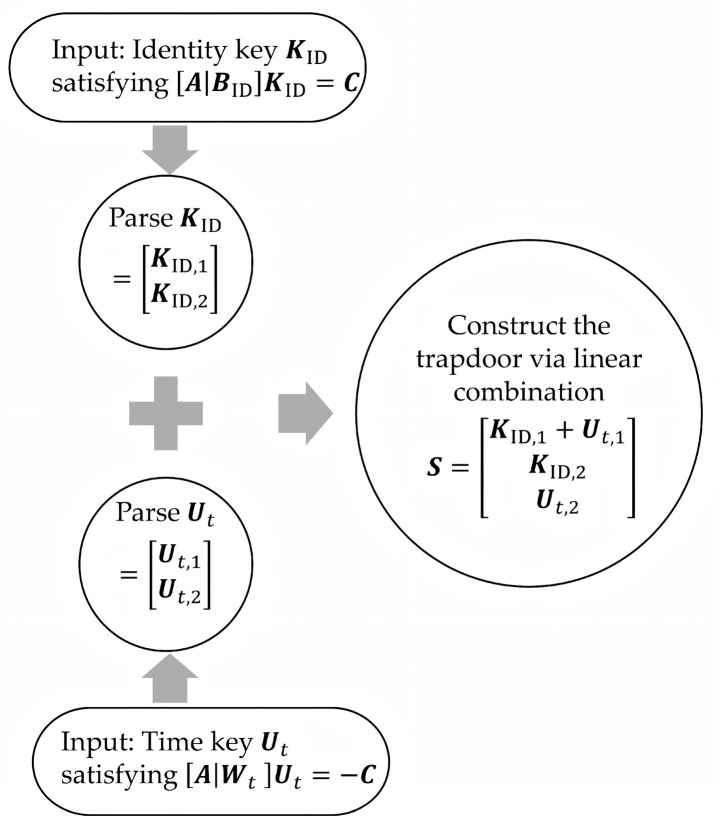
Dual-key combination trapdoor generation mechanism.

As shown in Figure 3, the specific procedural details are as follows:

Firstly, KGC generates an identity key $K_{ID}$ for each user, satisfying $\left[A|B_{ID}\right]K_{ID}=C$. At this point, the identity key $K_{ID}$ is no longer a trapdoor for $\left[A|B_{ID}\right]\;$ and cannot be directly used to solve the short vectors of other instances.

Secondly, in each time period, KGC computes time keys $\;U_{t}$ for non-revoked users satisfying $\left[A|W_{t}\right]U_{t}=-C$, where $W_{t}$ is a matrix representing the time period.

Finally, non-revoked users perform a linear combination of their identity key $K_{ID}$ and time key $\;U_{t}.$ This operation corresponds to adding the two key equations, which yields:

$$\left[A|B_{ID}\right]K_{ID}+\left[A|W_{t}\right]U_{t}=0$$

Through simple matrix block operations and vector concatenation, this equation can be rewritten as:

ABID|WtKID,1+Ut,1KID,2Ut,2=0

At this step, the non-revoked user obtains a short basis for the lattice $\Lambda_{q}^{u}(A|B_{ID}|W_{t})$, the matrix ***S***=KID,1+Ut,1KID,2Ut,2.

The security of the proposed mechanism relies entirely on the computational hardness of the ISIS problem. Specifically, the core of its security hinges on the computational infeasibility of finding a short-norm solution vector ***s*** from the publicly available matrix $\left[A\left|B_{ID}\right|W_{t}\right]$ that satisfies the equation $\left[A\left|B_{ID}\right|W_{t}\right]\cdot s=c$.

The compromise of the ISIS problem would lead to a complete loss of confidentiality, as an adversary could derive valid decryption keys directly from public parameters—enabling decryption of any ciphertext for any identity across all time periods without requiring user-specific keys. Ultimately, such a scenario would trigger total system collapse, since the attack targets the cryptographic core rather than individual users, thereby invalidating all security guarantees and rendering the entire encryption scheme practically useless.

Based on the above mechanism, and drawing inspiration from the lattice-based delegation method in [6], we leverage the gadget matrix ***G*** to outsource the vector sampling operation during decryption key generation to untrusted servers, which further reduces the computational overhead for users. The scheme is constructed as follows:

- **Setup**. KGC generates a matrix ***A*** and its corresponding trapdoor $T_{A}$ using a trapdoor generation algorithm. Matrix ***A*** serves as a system’s public parameter, while the trapdoor $T_{A}$ serves as the system’s master secret key.

- **Secret Key Generation**. KGC inputs $T_{A}$ into the algorithm **SampleLeft** and generates a secret key $K_{ID}$ for each user. This key satisfies the equation $\left[A|B_{ID}\right]K_{ID}=G-C$. Here, ***C*** is a public parameter, $B_{ID}$ is a matrix representing the user identity, and ***G*** is the gadget matrix.

- **Key Update Generation**. In each time period, KGC inputs $T_{A}$ into the algorithm **SampleLeft**. Given the equation $\left[A|W_{t}\right]U_{t}=-C$, it generates and broadcasts key updates for non-revoked users. $W_{t}$ is a matrix representing the time period *t*.

- **Decryption Key Generation**. The user possesses the short basis ***S*, **which satisfies $\left[A|B_{ID}{|W}_{t}\right]S=0$. The receiver needs to generate the decryption key ${dk}_{ID,t}$ satisfying $[A|B_{ID}\left|W_{t}\right]{dk}_{ID,t}=u$, where ***u*** is a public parameter. The user accomplishes this by outsourcing the sampling of an intermediate vector ***x*** satisfying Gx=u to an untrusted server. After obtaining ***x***, the user computes ${dk}_{ID,t}=S\cdot x.$ This derivation is valid because $\left[A|B_{ID}{|W}_{t}\right]S\cdot x=G\cdot x=u$, which produces a valid short solution to the target equation.

## 5. Post-Quantum Secure, Lightweight RIBE Scheme with DKER

Parameters. We set the message space as $M=\left\{0,1\right\}$, the identity space as $ID\subset Z_{q}^{n}\backslash \{0_{n}\}$, and the time period space as $T\subset Z_{q}^{n}$. Typically, time periods are treated as natural numbers, and a hash function $H:N\to Z_{q}^{n}$ is used to map natural numbers into vector form. For any $B\in N,\;\;U_{B}$ denotes the uniformly random distribution on Z∩[−B,B]. Moreover, the selection of our security parameters is constrained by the following requirements: $m>2nlog\;q,\;\;\sigma >\sqrt{m}\cdot \omega \left(\sqrt{m}\right),n=O\left(\lambda \right),{\;\chi }_{LWE}=D_{Z,\sigma },\chi_{big}=U_{B},B>(m\sigma^{2}+1)2^{\lambda }$.

### 5.1. Setup

Input a security parameter λ and the maximum number of system users *N*. Output the system public parameter PP and master secret key MSK.

1. Run the trapdoor generation algorithm $TrapGen\left(n,q\right)\;$to generate a matrix $A\in Z_{q}^{n\times m}$ and its corresponding trapdoor $T_{A}\in Z^{m\times m}$.
2. Select uniformly random matrices ***B***, ***W***$⟵Z_{q}^{n\times m}$, and select an *n*-dimensional vector $u⟵Z_{q}^{n}$ uniformly at random.
3. Create a binary tree BT containing at least *N *leaf nodes. Mark all leaf nodes as unassigned. For each node θ in the binary tree, select a uniformly random matrix $C_{\theta }⟵Z_{q}^{n\times m}.$
4. Output the system public parameters $PP=\left\{A,B,W,\left\{C_{\theta }|\theta \in BT\right\},u\right\}$ and the master secret key $MSK=\left\{T_{A}\right\}$.

### 5.2. GenSK

Input the system public parameters PP, the master secret key MSK, and a user identity $ID\in Z_{q}^{n}$. Output the secret key ${SK}_{ID}$ corresponding to identity ID.

1. Randomly select an unassigned leaf node η in the binary tree BT, and store the identity ID in this leaf node. Let $\eta_{ID}\;$denote the leaf node storing identity ID. Let $Path\left(\eta_{ID}\right)$ denote the set of all nodes on the path from the leaf node $\eta_{ID}$ to the root node (including both the leaf and the root).
2. Let $B_{ID}=B+H(ID)G$, where the hash function H is defined in Definition 1, and ***G*** is the gadget matrix in Lemma 3.
3. For the identity ID and each node θ in $Path\left(\eta_{ID}\right)$, generate $K_{ID,\theta }\;$satisfying the equation $\left[A|B_{ID}\right]K_{ID,\theta }=G-C_{\theta }$, as follows:
  - Select a uniformly random matrix $K_{ID}^{\prime }\leftarrow \chi_{LWE}^{2m\times m}$. Compute $Z_{ID}=[A|B_{ID}]K_{ID}^{\prime }$.
  - Sample $K_{ID,\theta }^{\prime \prime }\leftarrow SampleLeft\left(A,B_{ID},T_{A},G-C_{\theta }-Z_{ID},\sigma \right)$.
  - Split $K_{ID}^{\prime }$ into two parts, the first m rows denoted as $K_{ID,1}^{\prime }$, and the last m rows denoted as $K_{ID,2}^{\prime }$. Similarly, split $K_{ID,\theta }^{\prime \prime }$ into $K_{ID,\theta ,1}^{\prime \prime }$ and $K_{ID,\theta ,2}^{\prime \prime }$. Construct the key update matrix as: $K_{ID,\theta }={\left[\left(K_{ID,1}^{\prime }+K_{ID,\theta ,1}^{\prime \prime }\right)|{(K}_{ID,2}^{\prime }+K_{ID,\theta ,2}^{\prime \prime })\right]}^{T}\in Z_{q}^{2m\times m}$.
4. Output the secret key ${SK}_{ID}=\{(\theta ,K_{ID,\theta })|\theta \in Path\left(\eta_{ID}\right)\}$.

### 5.3. KeyUp

Input the system public parameters PP, the master secret key MSK, the revocation list ${RL}_{t}$ for time period *t*, and the binary tree BT. Output the key update ${KU}_{t}$.

1. Run the **KUNode** algorithm, inputting the binary tree BT and the revocation list ${RL}_{t}$ for time period *t*. This outputs the node update set $NodeUP\left({RL}_{t}\right)$ for time period *t*.
2. Compute $W_{t}=W+H(t)G$, where the hash function H is defined in Definition 1, and ***G*** is the gadget matrix from Lemma 3.
3. For each node in the $NodeUP\left({RL}_{t}\right)$, generate a key update$\;T_{\theta ,t}$ satisfying the equation $\left[A|W_{t}\right]T_{\theta ,t}=C_{\theta }$, as follows:
  - Select a uniformly random matrix $\;T_{t}^{\prime }\leftarrow \chi_{LWE}^{2m\times m}$. Compute $Y_{t}=\left[A|W_{t}\right]T_{t}^{\prime }$.
  - Sample $T_{\theta ,t}^{\prime \prime }\leftarrow SampleLeft\left(A,W_{t},T_{A},C_{\theta }-Y_{t},\sigma \right)$.
  - Split $T_{t}^{\prime }$ into two parts, the first *m *rows denoted as $\;T_{t,1}^{\prime }$, and the last *m *rows denoted as $T_{t,2}^{\prime }$. Similarly, split $T_{\theta ,t}^{\prime \prime }$ into $T_{\theta ,t,1}^{\prime \prime }$ and $T_{\theta ,t,2}^{\prime \prime }$. Construct the key update matrix as:
    $$T_{\theta ,t}={\left[\left(T_{t,1}^{\prime }+T_{\theta ,t,1}^{\prime \prime }\right)|(T_{t,2}^{\prime }+T_{\theta ,t,2}^{\prime \prime })\right]}^{T}\in Z_{q}^{2m\times m}$$
4. Broadcast the key update ${KU}_{t}=\{(\theta ,T_{\theta ,t})|\theta \in NodeUP\left({RL}_{t}\right)\}$.

### 5.4. Enc

Input the system public parameters PP, a message μ⟵{0,1}, an identity $\;ID\in Z_{q}^{n}$, and a time period $t\in Z_{q}^{n}$. Output ciphertext ${CT}_{ID,t}$.

1. Select uniformly random matrices ***R***, ***V***$⟵{\left\{-1,1\right\}}^{m\times m}$, and select a uniformly random vector $s\leftarrow Z_{q}^{n}$.
2. Sample noise $e_{0}⟵\chi_{LWE}$, and sample an *m*-dimensional noise vector $e_{1}\leftarrow \chi_{LWE}^{m}$.
3. Compute: $c_{0}=su^{T}+e_{0}+\left\lfloor \frac{q}{2}\right\rfloor \mu$, $c_{ID,t}=s\left[A|B_{ID}|W_{t}\right]+e_{1}[I_{m}|R|V]$, where $I_{m}$ is an identity matrix.
4. Output the ciphertext ${CT}_{ID,t}=(c_{0},{\;c}_{ID,t})$.

### 5.5. GenDK

Input system public parameters PP, the secret key ${SK}_{ID}$ corresponding to the identity ID, and the key update ${KU}_{t}$ for time period *t*. Output the decryption key ${DK}_{ID,t}$.

1. Compare the binary tree nodes associated with their secret key against the nodes in the key update ${KU}_{t}\;$for time period *t*. If a common node exists, denote this node as $\theta^{*}$ and proceed with the following steps. Otherwise, the algorithm aborts.
2. Select $K_{ID,\theta^{*}}\;$from their secret key ${SK}_{ID}$ and $T_{\theta^{*},t}$ from the key update ${KU}_{t}$. Generate the decryption key ${dk}_{ID,t}$ satisfying the equation $[A\left|B_{ID}\right|W_{t}]{{dk}_{ID,t}}^{T}=u^{T}$.
  - Select a vector $k_{t}\leftarrow \chi_{big}^{3m}$ and compute$\;{h_{ID,t}}^{T}=\;[A\left|B_{ID}\right|W_{t}]{k_{t}}^{T}$.
  - Sample ${k_{ID,t}^{\prime }}^{T}\leftarrow SamplePre\left(G,T_{G},u-h_{ID,t},\sigma \right)$.
  - Construct the combined matrix ***S*** using the secret key component $\;K_{ID,\theta^{*}}$ and the key update component $T_{\theta^{*},t}$: $S={\;[\left(K_{ID,1}^{\prime }+K_{ID,\theta^{*},1}^{\prime \prime }+T_{t,1}^{\prime }+T_{\theta^{*},t,1}^{\prime \prime }\right)\left|{(K}_{ID,2}^{\prime }+K_{ID,\theta^{*},2}^{\prime \prime })\right|(T_{\theta^{*},t,2}^{\prime \prime }+T_{t,2}^{\prime })]}^{T}\in Z_{q}^{3m\times m}$.
  - Compute ${K_{ID,\theta^{*},t}^{\prime \prime }}^{T}=S\cdot k_{ID,t}^{\prime }$.
  - Split the vector $k_{t}$ into three blocks of *m *elements each, denoted as $k_{t,1}$, $k_{t,2}$, and $k_{t,3}$. Split the resulting vector ${K_{ID,\theta^{*},t}^{\prime \prime }}^{T}$ into three blocks of *m *elements each, denoted as ${K_{ID,\theta^{*},t,1}^{\prime \prime }}^{T},{K_{ID,\theta^{*},t,2}^{\prime \prime }}^{T}$ and ${K_{ID,\theta^{*},t,3}^{\prime \prime }}^{T}$. Construct the final decryption key vector:
    $${dk}_{ID,t}=\;[(k_{t,1}+{K_{ID,\theta^{*},t,1}^{\prime \prime }}^{T})\left|\left(k_{t,2}+{K_{ID,\theta^{*},t,2}^{\prime \prime }}^{T}\right)\right|{(k}_{t,3}+{K_{ID,\theta^{*},t,3}^{\prime \prime }}^{T})]{\in Z}_{q}^{3m}$$
3. Output decryption key ${DK}_{ID,t}={dk}_{ID,t}$.

### 5.6. Dec

Input the system public parameters PP, the ciphertext ${CT}_{ID,t}$, and the decryption key ${DK}_{ID,t}$. Output message μ.

1. Compute $c^{\prime }=c_{0}-c_{ID,t}\cdot {{dk}_{ID,t}}^{T}$. If $\left|c^{\prime }-\left\lfloor \frac{q}{2}\right\rfloor \right|<\left\lfloor \frac{q}{4}\right\rfloor$ then μ=1; otherwise μ=0.
2. Output the message μ.

## 6. Performance Comparison

### 6.1. Correctness

Next, we analyze the correctness of the proposed scheme,

$$c^{\prime }=c_{0}-c_{ID,t}\cdot {{dk}_{ID,t}}^{T}=\left\lfloor \frac{q}{2}\right\rfloor \mu +e_{0}-e_{1}[I_{m}|R|V]{{dk}_{ID,t}}^{T}$$

The error term in the decryption process is $e_{0}-e_{1}[I_{m}|R|V]{{dk}_{ID,t}}^{T}$.

$$e_{0}-e_{1}\;[I_{m}\left|R\right|V]{{dk}_{ID,t}}^{T}=e_{0}-e_{1}\;[I_{m}|R|V]\left[\begin{array}{c} k_{t,1}+\left(K_{ID,1}^{\prime }+K_{ID,\theta^{*},1}^{\prime \prime }+T_{t,1}^{\prime }+T_{\theta^{*},t,1}^{\prime \prime }\right)k_{ID,t,1}^{\prime }\\ k_{t,2}+\left(K_{ID,2}^{\prime }+K_{ID,\theta^{*},2}^{\prime \prime }\right)k_{ID,t,2}^{\prime }\\ k_{t,3}+(T_{\theta^{*},t,2}^{\prime \prime }+T_{t,2}^{\prime })k_{ID,t,3}^{\prime } \end{array}\right]$$

According to Lemma 1, $\left|\left|R\right|\right|,||V||\leq O(\sqrt{m})$.

According to Lemma 3, $\left|\left|k_{t,1}\right|\right|,\left|\left|k_{t,2}\right|\right|,\left|\left|k_{t,3}\right|\right|,||k_{t,4}||\leq \sqrt{m}B$.

According to Lemma 3, $\left|\left|\left(K_{ID,1}^{\prime }+K_{ID,\theta^{*},1}^{\prime \prime }+T_{t,1}^{\prime }+T_{\theta^{*},t,1}^{\prime \prime }\right)k_{ID,t,1}^{\prime }\right|\right|,\left|\left|\left(K_{ID,2}^{\prime }+K_{ID,\theta^{*},2}^{\prime \prime }\right)\;k_{ID,t,2}^{\prime }\right|\right|,\left|\left|(T_{\theta^{*},t,2}^{\prime \prime }+T_{t,2}^{\prime })\;k_{ID,t,3}^{\prime }\right|\right|\leq m^{3/2}\sigma$, and similarly for other terms of this matrix.

According to Lemma 3, $\left|\left|e_{0}\right|\right|\leq \sigma ,||e_{1}||\leq \sqrt{m}\sigma$. Therefore, this analysis leads to the following upper bound for the error term:

$$\left|\left|e_{0}-e_{1}\left[I_{m}|R|V\right]{{dk}_{ID,t}}^{T}\right|\right|\leq \left|\left|e_{0}\right|\right|+||e_{1}||\cdot ||\left[I_{m}|R|V\right]{{dk}_{ID,t}}^{T}||\;\;\;\;\;\;\;\;\;\;\;\;\;\;\;\;\;\;\;\;\;\;\;\;\;\;\;\;\;\;\;\;\;\;\;\;\;\;\;\;\;\;\;\;\;\;\;\;\;\;\leq \sigma +\sqrt{m}\sigma \left[m^{3/2}\sigma +3\sqrt{m}B\right]O\left(\sqrt{m}\right)\;\;\;\;\;\;\;\;\;\;\;\;\;\;\;\;\;\;\;\;\;\;\;\;\leq O\left(m^{3/2}B\sigma \right)<q/4.$$

When the parameters in the scheme satisfy the relation $O\left(m^{3/2}B\sigma \right)<q/4\;$ the scheme can successfully decrypt and recover the plaintext.

### 6.2. Security Analysis

**Theorem** **1.** *Under the decision-LWE and ISIS hardness assumptions, the proposed PQS-LRIBE-DKER scheme is provably secure in the IND-sID-CPA security model.*

**Proof.**  The selective identity security is proved through a series of games. The first game in this sequence is indistinguishable from the real IND-sID-CPA. In the final game of the sequence, the adversary’s advantage is zero. Since any probabilistic polynomial-time adversary cannot distinguish any two consecutive games in the sequence, the adversary’s advantage in winning the original IND-sID-CPA game is negligible. In conclusion, under the decision-LWE and ISIS hardness assumptions, the proposed scheme possesses selective identity security.

**Game 0:** The adversary A interacts with the challenger C in the original IND-sID-CPA game as defined by the security model.

**Game 1:** Building upon Game 0, the challenger C generates matrices ***B*** and ***W*** in the following manner during the setup phase. Let ${ID}^{*}$ and $\;t^{*}$ denote the challenge identity and challenge time period chosen by the adversary A. The challenger C moves the step of selecting matrices $R^{*}$ and $V^{*}$ forward to the setup phase, sampling uniformly random matrices $R^{*},V^{*}\leftarrow {\left\{-1,1\right\}}^{m\times m}$. Then, compute the following:

$$B=AR^{*}-H\left({ID}^{*}\right)G$$

$$W=AV^{*}-H\left(t^{*}\right)G$$

**Reduction from Game 0 to Game 1:** According to Lemma 1, if a matrix ***A***${\in Z}_{q}^{n\times m}$ is selected uniformly at random, and a matrix $R{\in \left\{-1,1\right\}}^{m\times m}$ is uniformly random, then the distribution of (***A***, ***AR***) is statistically indistinguishable from the distribution of (***A***, ***E***), where ***E*** is a uniformly random matrix in $\;Z_{q}^{n\times m}$. Therefore, in Game 1, the distribution of matrix $B=AR^{*}-H\left({ID}^{*}\right)G$ is statistically indistinguishable from the distribution of ***E*** to the adversary A. The same applies to matrix ***W***. Consequently, Game 0 and Game 1 are indistinguishable to the adversary A.

**Game 2:** Based on Game 1, the challenger C changes the way of responding to secret key queries.

For a Type I adversary, challenger C needs to respond to queries for the secret key ${SK}_{{ID}^{*}}$ corresponding to the challenge identity. During the setup phase, set $C_{\theta }=G-\left[A|AR^{*}\right]K_{{ID}^{*},\theta },\;\theta \in Path\left(\eta_{{ID}^{*}}\right)$.

1. $ID={ID}^{*}$.
  For $\theta \in Path\left(\eta_{{ID}^{*}}\right)$, select $K_{{ID}^{*},\theta }\leftarrow \chi_{LWE}^{2m\times m}$ and compute $C_{\theta }=G-\left[A|AR^{*}\right]K_{{ID}^{*},\theta }$.
2. $ID\neq {ID}^{*}$.
  For $\theta \in Path\left(\eta_{{ID}^{*}}\right)\cap Path\left(\eta_{ID}\right)$, compute $C_{\theta }=G-\left[A|AR^{*}\right]K_{{ID}^{*},\theta }$.
  - Select $K_{ID}^{\prime }\leftarrow \chi_{LWE}^{2m\times m}$ and compute $Z_{ID}=[A|B_{ID}]K_{ID}^{\prime }$.
  - Sample $K_{ID,\theta }^{\prime \prime }\leftarrow SampleRight(A,AR^{*}+H\left(ID\right)-H\left({ID}^{*}\right),G,T_{G},G-C_{\theta }-Z_{ID},\sigma )$.
  For $\theta \in Path\left(\eta_{ID}\right)/Path\left(\eta_{{ID}^{*}}\right)$, select uniformly random matrices $C_{\theta }⟵Z_{q}^{n\times m}$.
  - Select $K_{ID}^{\prime }\leftarrow \chi_{LWE}^{2m\times m}$ and compute $Z_{ID}=\left[A|B_{ID}\right]K_{ID}^{\prime }$.
  - Sample $K_{ID,\theta }^{\prime \prime }\leftarrow SampleRight(A,AR^{*}+H\left(ID\right)-H\left({ID}^{*}\right),G,T_{G},G-C_{\theta }-Z_{ID},\sigma )$.

For a Type II adversary, challenger C responds to secret key queries except challenge identity ${ID}^{*}$. In the setup phase, select uniformly random matrices$\;C_{\theta }⟵Z_{q}^{n\times m}$.

1. ID ≠ ID^∗^.
  - Select $K_{ID}^{\prime }\leftarrow \chi_{LWE}^{2m\times m}$ and compute $Z_{ID}=\;[A|B_{ID}]K_{ID}^{\prime }$.
  - Sample $K_{ID,\theta }^{\prime \prime }\leftarrow SampleRight(A,AR^{*}+H\left(ID\right)-H\left({ID}^{*}\right),G,T_{G},G-C_{\theta }-Z_{ID},\sigma )$.

**Reduction from Game 1 to Game 2:** In Game 1, the matrices $C_{\theta }\leftarrow Z_{q}^{n\times m}\;$ are selected uniformly at random. In Game 2, the matrices $C_{\theta }\;$ are defined as $C_{\theta }=G-\left[A|AR^{*}\right]K_{{ID}^{*},\theta }\;,$ where $\theta \in Path\left(\eta_{{ID}^{*}}\right)$. According to the ISIS assumption, the output of the preimage sampleable function is uniformly random. Therefore, the matrices $\;C_{\theta }$ in Game 1 and Game 2 are indistinguishable to the adversary A. Furthermore, in Game 1, the matrices $K_{ID,\theta }^{\prime \prime }$ are generated using the **SampleLeft** algorithm. In Game 2, the challenger samples the matrices $K_{ID,\theta }^{\prime \prime }$ using the **SampleRight** algorithm. According to Lemma 3, the statistical distance between the outputs of the **SampleLeft** and **SampleRight** algorithms is negligible, and the output of the **SampleRight** algorithm is computationally indistinguishable from the $\chi_{LWE}$. Consequently, Game 1 and Game 2 are also indistinguishable to the adversary A.

**Game 3:** Building upon Game 2, the challenger C modifies the method for responding to key update queries.

For a Type I adversary, while the adversary A is permitted to query the secret key ${SK}_{{ID}^{*}}$ corresponding to the challenge identity, according to the security model definition, the adversary A is subsequently prohibited from querying the key update corresponding to the challenge time period.

1. $t\neq t^{*}$.
  - Select $T_{t}^{\prime }\leftarrow \chi_{LWE}^{2m\times m}$ and set $Y_{t}=\;[A|AV^{*}]T_{t}^{\prime }$.
  - Sample $T_{\theta ,t}^{\prime \prime }\leftarrow SampleRight(A,V^{*},G,T_{G},H\left(t\right)-H\left(t^{*}\right),C_{\theta }-Y_{t},\sigma )$.

For a Type II adversary, the challenger C must respond to the adversary’s query for the key update $\;T_{\theta ,t^{*}}$ corresponding to the challenge time $t^{*}$. The matrix $C_{\theta }$ is set as $C_{\theta }=\left[A|AV^{*}\right]T_{\theta ,t^{*}}\;,\;\theta \in Path\left(\eta_{{ID}^{*}}\right)$.

1. $t=t^{*}$.
  For $\theta \in Path\left(\eta_{{ID}^{*}}\right)$, select $T_{\theta ,t^{*}}\leftarrow \chi_{LWE}^{2m\times m}$ and return $T_{\theta ,t^{*}}$ to the adversary A.
  For $\theta \notin Path\left(\eta_{{ID}^{*}}\right)$,
  - Select $T_{t}^{\prime }\leftarrow \chi_{LWE}^{2m\times m}$ and set $Y_{t}=\;[A|AV^{*}]T_{t}^{\prime }$.
  - Sample $T_{\theta ,t}^{\prime \prime }\leftarrow SampleRight(A,V^{*},G,T_{G},H\left(t\right)-H\left(t^{*}\right),C_{\theta }-Y_{t},\sigma )$.
2. $t\neq t^{*}$.
  - Select $T_{t}^{\prime }\leftarrow \chi_{LWE}^{2m\times m}$ and set $Y_{t}=\;[A|AV^{*}]T_{t}^{\prime }$.
  - Sample $T_{\theta ,t}^{\prime \prime }\leftarrow SampleRight(A,V^{*},G,T_{G},H\left(t\right)-H\left(t^{*}\right),C_{\theta }-Y_{t},\sigma )$.

**Reduction from Game 2 to Game 3:** The difference between Game 2 and Game 3 lies in the method of generating key updates. In Game 2, the matrix $T_{\theta ,t}^{\prime \prime }$ is generated using the **SampleLeft** algorithm. In Game 3, the challenger selects the matrix $T_{\theta ,t^{*}}$ from the $\chi_{LWE}^{2m\times m}$ obtaining it by sampling using the **SampleRight** algorithm. According to Lemma 3, the statistical distance between the outputs of the **SampleLeft** and **SampleRight** algorithms is negligible, and the output of the **SampleRight** algorithm is computationally indistinguishable from the $\chi_{LWE}$ distribution. Therefore, Game 2 and Game 3 are also indistinguishable to the adversary A.

**Game 4:** Building upon Game 3, the challenger C modifies the method of generating decryption keys.

For a Type I adversary, when $\;{ID\neq ID}^{*},t=t^{*}$, and $\;\theta \in Path\left(\eta_{{ID}^{*}}\right)$, the challenger cannot simulate the key update $T_{\theta ,t^{*}}$. Challenger C responds to decryption key queries for ${dk}_{ID,t^{*}}$ according to the following steps:

1. Select a matrix $T_{\theta ,t^{*}}\leftarrow \chi_{LWE}^{2m\times m}$ and compute $K=\left[A|AV^{*}\right]T_{\theta ,t^{*}}$.
2. Solve for the secret key $K_{ID,\theta }$ satisfying the equation $\left[A|AR^{*}+(H\left(ID\right)-H\left({ID}^{*}\right))\right]K_{ID,\theta }=G-K$.
  - Select a matrix $K_{ID}^{\prime }\leftarrow \chi_{LWE}^{2m\times m}\;$and compute $Z_{ID}=\left[A|AR^{*}+(H\left(ID\right)-H\left({ID}^{*}\right))G\right]K_{ID}^{\prime }$.
  - Sample $K_{ID,\theta }^{\prime \prime }\leftarrow SampleRight(A,AR^{*},G,T_{G},H\left(ID\right)-H\left({ID}^{*}\right),G-Z_{ID}-K,\sigma )$.
3. Compute ${dk}_{ID,t^{*}}$ using $K_{ID,\theta }$ and $T_{\theta ,t^{*}}$.
  - Select a vector $k_{t}\leftarrow \chi_{big}^{3m}$ and set ${h_{ID,t}}^{T}=\;[A\left|B_{ID}\right|W_{t}]{k_{t}}^{T}$.
  - Sample ${k_{ID,t}^{\prime }}^{T}\leftarrow SamplePre\left(G,T_{G},u-h_{ID,t},\sigma \right)$.
  - Compute $\;S={\;[\left(K_{ID,1}^{\prime }+K_{ID,\theta^{*},1}^{\prime \prime }+T_{t,1}^{\prime }+T_{\theta^{*},t,1}^{\prime \prime }\right)\left|{(K}_{ID,2}^{\prime }+K_{ID,\theta^{*},2}^{\prime \prime })\right|(T_{\theta^{*},t,2}^{\prime \prime }+T_{t,2}^{\prime })]}^{T}\in Z_{q}^{3m\times m}$ using$\;K_{ID,\theta^{*}}\;and\;T_{\theta^{*},t}$.
  - Compute ${K_{ID,\theta^{*},t}^{\prime \prime }}^{T}=S\cdot k_{ID,t}^{\prime }$.
  - Perform row blocking on the vector $k_{t}$, dividing it into three blocks of *m *rows each, denoted as $k_{t,1}$, $k_{t,2}$, and $k_{t,3}$. Perform row blocking on the ${K_{ID,\theta^{*},t}^{\prime \prime }}^{T}$, dividing it into three blocks of *m *rows each, denoted as $\;{K_{ID,\theta^{*},t,1}^{\prime \prime }}^{T},{K_{ID,\theta^{*},t,2}^{\prime \prime }}^{T}$, and ${K_{ID,\theta^{*},t,3}^{\prime \prime }}^{T}$. Construct the final decryption key vector by adding the corresponding blocks:
    $${dk}_{ID,t}=\;[(k_{t,1}+{K_{ID,\theta^{*},t,1}^{\prime \prime }}^{T})\left|\left(k_{t,2}+{K_{ID,\theta^{*},t,2}^{\prime \prime }}^{T}\right)\right|{(k}_{t,3}+{K_{ID,\theta^{*},t,3}^{\prime \prime }}^{T})]{\in Z}_{q}^{3m}$$
4. Output decryption key $\;{DK}_{ID,t}={dk}_{ID,t}$.

For a Type II adversary, when ${ID=ID}^{*},t\neq t^{*}$, and $\;\theta \in Path\left(\eta_{{ID}^{*}}\right)$, the challenger cannot simulate the secret key $K_{{ID}^{*},\theta }$. The challenger responds to decryption key queries for $\;{dk}_{{ID}^{*},\;\;t}$ according to the following steps:

1. Select a matrix $K_{{ID}^{*},\theta }\leftarrow \chi_{LWE}^{2m\times m}$ and let $K=\left[A|AR^{*}\right]K_{{ID}^{*},\theta }$.
2. Next, solve for the key update $T_{\theta ,t}$ satisfying the equation $\left[A|AV^{*}+(H\left(t\right)-H\left(t^{*}\right))G\right]T_{\theta ,t}=G-K$.
  - Select a matrix $T_{t}^{\prime }\leftarrow \chi_{LWE}^{2m\times m}\;$and compute $Y_{t}=\left[A|AV^{*}+(H\left(t\right)-H\left(t^{*}\right))G\right]T_{t}^{\prime }$.
  - Sample $T_{\theta ,t}^{\prime \prime }\leftarrow SampleRight(A,V^{*},G,T_{G},H\left(t\right)-H\left(t^{*}\right),G-Y_{t}-K,\sigma )$.
3. Compute the decryption key using $K_{{ID}^{*},\theta }$ and $T_{\theta ,t}$. The remaining steps are the same as in the scheme.

**Reduction from Game 3 to Game 4:** The difference between Game 4 and Game 3 exists in the method of generating decryption keys. In Game 3, the key update consists of a matrix $T_{t}^{\prime }\leftarrow \chi_{LWE}^{2m\times m}$ and a matrix $T_{\theta ,t}^{\prime \prime }\;$sampled via the **SampleRight** algorithm; the secret key consists of a matrix $K_{ID}^{\prime }\leftarrow \chi_{LWE}^{2m\times m}$ and a matrix $K_{ID,\theta }^{\prime \prime }$. In Game 4, the key update is $T_{\theta ,t^{*}}\leftarrow \chi_{LWE}^{2m\times m}$ and the secret key is $K_{{ID}^{*},\theta }\leftarrow \chi_{LWE}^{2m\times m}$. For the adversary A, the $\chi_{LWE}$ distribution is computationally indistinguishable from the output of the **SampleRight** algorithm. In the end, the decryption keys derived from the secret key and key update are indistinguishable for the adversary A. Game 3 and Game 4 are also indistinguishable to the adversary A.

**Game 5**: Building upon Game 4, the challenger C modifies the generation methods for matrices ***A***, ***B,*** and ***W***. In Game 4, the challenger C uses the **TrapGen** algorithm to generate matrix ***A*** and its corresponding trapdoor $T_{A}$, while selecting matrices ***B*** and ***W*** uniformly at random. In Game 5, the challenger selects the uniformly random matrix $A\leftarrow Z_{q}^{n\times m}$ and uses the **TrapGen** algorithm to generate matrices ***B***, ***W***, and their corresponding trapdoors.

**Game 6:** Building upon Game 5, the challenger C modifies the method for generating the challenge ciphertext. In Game 6, the challenger C randomly selects $c_{0}\leftarrow Z_{q}$ and ${\;c}_{ID,t}\leftarrow Z_{q}^{3m}$ as the challenge ciphertext.

**Reduction from Game 5 to Game 6:** If the adversary A can distinguish between Game 5 and Game 6, then the simulator B can leverage the adversary A to solve the decision-LWE problem.

Assume the adversary A can distinguish between Game 5 and Game 6 with a non-negligible probability. Then the simulator B utilizes the adversary A to solve the decision-LWE problem defined in Definition 2. The reduction process is as follows:

**Instance:** Given the decision-LWE instance ${\left\{u_{i},v_{i}\right\}}_{i\in \;[m]}\in Z_{q}^{n}\times Z_{q}$ to simulator B. The task for the simulator B is to distinguish whether the $v_{i}$ are generated by $v_{i}=u_{i}s^{T}+e_{i}\left(e_{i}\leftarrow \chi_{LWE}\right)\;$ or are chosen from a uniformly random distribution.

**Init:** The adversary A sends the challenge identity ${ID}^{*}$ and challenge time period $t^{*}$ to the simulator BB.

**Setup:** In the setup phase, the simulator B constructs the matrix $A=\left(u_{1}^{T}|\ldots |u_{m}^{T}\right){\in Z}_{q}^{n\times m}$ and sets $u=u_{0}$.

**Query Phase:** The adversary A makes secret key queries, key update queries, decryption key queries, and revocation queries. The simulator B responds to these queries according to the method used in Game 5.

**Challenge Phase:** The adversary A sends the plaintext messages $\left(m_{0},m_{1}\right)$ to the simulator B.

The simulator B generates the challenge ciphertext:

1. Let $v=(v_{1}|\ldots |v_{m})$.
2. Compute $c^{*}=v_{0}+\left\lfloor \frac{q}{2}\right\rfloor m_{b},c_{ID,t}^{*}=v[I_{m}|R^{*}|V^{*}]$. Send the challenge ciphertext ${ct}^{*}=(c^{*},c_{ID,t}^{*})$ to the adversary A.

**Guess Phase:** After receiving the challenge ciphertext, the adversary A makes a guess and returns b’. The simulator B uses the adversary’s guess $b^{\prime }$ as the answer to the decision-LWE problem.

**Analysis:**

- When $v_{i}=u_{i}s^{T}+e_{i}$, the challenge ciphertext is:
  $$c_{0}=v_{0}+\left\lfloor \frac{q}{2}\right\rfloor m_{b}=us^{T}+e_{0}+\left\lfloor \frac{q}{2}\right\rfloor m_{b}$$
  $${\;c}_{ID,t}=v\left[I_{m}|R^{*}|V^{*}\right]=\left(sA+e\right)\left[I_{m}|R^{*}|V^{*}\right]=s\left[A|B_{{ID}^{*}}|W_{t^{*}}\right]+e\left[I_{m}|R^{*}|V^{*}\right]$$

Therefore, when $v_{i}=u_{i}s^{T}+e_{i}$, the distribution of the challenge ciphertext is identical to that in Game 5.

- When the ${(u}_{i},v_{i})$ are uniformly random elements, the distribution of the challenge ciphertext is identical to that in Game 6.

Consequently, the advantage of the simulator B for the decision-LWE problem equals the distinguishing advantage of the adversary A between Game 5 and Game 6. Given that the simulator’s advantage is negligible, the adversary A’s advantage must likewise be negligible. □

### 6.3. Comparison of Complexity

Table 1 presents a comparison of the proposed PQS-LRIBE-DKER scheme with other RIBE schemes with DKER across multiple key performance metrics. Specifically, KGC periodic workload refers to the computational effort required for KGC to execute undo operations within each time period; the user computational overhead includes the computational cost required to generate ciphertext during the encryption process.

In terms of security, both the Scalable RIBE with DKER scheme [5] and the Efficient RIBE with DKER scheme [25] are constructed based on traditional mathematical hard problems and cannot resist attacks from quantum computers. The LB-RIBE with B-DKER scheme [15] has an upper limit on the number of decryption key leaks, making it a bounded decryption key exposure resistance scheme.

For the LB-RHIBE with DKER scheme [16], users sample short vectors when generating decryption keys. Compared to the aforementioned schemes, the proposed PQS-LRIBE-DKER scheme enables the sampling process during decryption key generation to be outsourced to an untrusted third party, only needing to perform some simple matrix multiplication operations locally. In the LB-RIBE with En-DKER scheme [6], the sender needs to generate O(rlog(N/r)) ciphertexts for a single plaintext message, while in the OO-IRIBE-EnDKER scheme [17], the number of ciphertexts is O(N−r). The revocation in the LB-SA-RIBE scheme [26] requires a semi-trusted third-party server.

Table 2 presents a comparison between the PQS-LRIBE-DKER scheme and other lattice-based RIBE with DKER schemes, focusing on secret key length, key update length, decryption key length, and ciphertext length. Here, ciphertext length refers to the total length of the ciphertext generated by encrypting a single bit, while the parameters *n *and *m *denote the number of rows and columns of the matrix, respectively.

As shown in Table 2, the LB-RIBE with B-DKER scheme [15] achieves shorter key and ciphertext lengths, albeit with weaker security. The proposed PQS-LRIBE-DKER scheme offers multiple advantages: it employs a shorter decryption key than the LB-RHIBE with DKER scheme [16]; meanwhile, it also generates a shorter ciphertext length when encrypting a single bit compared to both the LB-RIBE with En-DKER scheme [6] and the OO-IRIBE-EnDKER scheme [17].

Table 3 compares the theoretical workload of users in our scheme with prior schemes across two dimensions: decryption key generation and ciphertext generation.

During the decryption key generation phase, users must execute the preimage sampling algorithm, which is the most computationally intensive and time-consuming operation in lattice-based cryptography, with a typical complexity of O(mnlogq). Therefore, we use the number of invocations of the preimage sampling algorithm to measure the user’s workload for generating decryption keys. As shown in Table 3, both the LB-RIBE with B-DKER scheme [15] and the LB-RHIBE with DKER scheme [16] require users to perform preimage sampling once per time period to generate decryption keys. In contrast, the LB-RIBE with En-DKER scheme [6], OO-IRIBE-EnDKER scheme [17], and our proposed PQS-LRIBE-DKER scheme allow users to offload the sampling tasks to untrusted third-party servers, thereby completely avoiding this overhead.

In terms of ciphertext generation, the user’s workload is characterized by the number of ciphertexts produced. Table 3 shows that in the LB-RIBE with En-DKER scheme [6], encrypting a single plaintext message requires generating $O\left(r\log\left(N/r\right)\right)$ ciphertexts; in the OO-IRIBE-EnDKER scheme [17], the number of ciphertexts is $O\left(N-r\right)$, whereas our scheme requires only one ciphertext. This single-ciphertext characteristic ensures that the encryption process remains highly efficient, and its performance does not degrade as the system scales. In summary, our scheme imposes the lightest computational burden on users.

## 7. Conclusions

This paper addresses the excessive computational and communication overhead in existing RIBE schemes with DKER. It proposes a lightweight RIBE scheme with DKER. We design a dual-key combination trapdoor generation mechanism, enabling non-revoked users to controllably derive decryption keys for the current time period by linearly combining identity keys and time keys. Furthermore, based on this dual-key combination trapdoor generation mechanism, the proposed scheme implements indirect revocation. This successfully shifts the periodic computational burden to a more computationally capable KGC, significantly reducing user computational costs and system communication overhead while enhancing overall system efficiency. We compare our scheme with other similar schemes, showing that the computational burden on the user in our scheme is lower than that in others, achieving a lightweight design for the user. A promising direction for future work is to investigate the applicability of this mechanism to more complex access control paradigms, particularly attribute-based encryption.

## Figures and Tables

**Table 1:** Comparison of Performance.

Scheme	Revocation Model	Post-Quantum Secure	Periodic Workload of KGC	Computational Cost of User
Scalable RIBE with DKER [5]	Indirect	F	O(rlog(N/r))	$O\left(1\right)$
Efficient RIBE with DKER [25]	Indirect	F	O(rlog(N/r))	$O\left(1\right)$
LB-RIBE with B-DKER [15]	Indirect	T	O(rlog(N/r))	$O\left(1\right)$
LB-RHIBE with DKER [16]	Indirect	T	O(rlog(N/r))	$O\left(1\right)$
LB-RIBE with En-DKER [6]	Direct	T	≈0	$\approx O\left(r\log\left(N/r\right)\right)+0$
LB-SA-RIBE [26]	Server-aided	T	O(rlog(N/r))	O(1)
OO-IRIBE-EnDKER [17]	Direct	T	0	$\approx O\left(N-r\right)+0$
PQS-LRIBE-DKER	Indirect	T	O(rlog(N/r))	$\approx O\left(1\right)+0$

**Table 2:** Comparison of Key Length and Ciphertext Length.

Scheme	Secret Key Length	Key Update Length	Decryption Key Length	Ciphertext Length
LB-RIBE with B-DKER [15]	2*m*	2*m*	4*m*	3*m *+ 1
LB-RHIBE with DKER [16]	3*m*	3*m*	6*m*	3*m *+ 1
LB-RIBE with En-DKER [6]	$3m^{2}$	0	4*m*	$O\left(r\log\left(N/r\right)\right)\cdot 4m+1$
OO-IRIBE-EnDKER [17]	$6m^{2}$	0	4*m*	$O\left(N-r\right)\cdot m\;$+ 3*m *+ 1
PQS-LRIBE-DKER	$2m^{2}$	$2m^{2}$	3*m*	3*m *+ 1

**Table 3:** Workload of Users.

Scheme	Decryption Key Generation	Ciphertext Generation
LB-RIBE with B-DKER [15]	$O\left(1\right)$	$O\left(1\right)$
LB-RHIBE with DKER [16]	$O\left(1\right)$	$O\left(1\right)$
LB-RIBE with En-DKER [6]	*0*	$O\left(r\log\left(N/r\right)\right)$
OO-IRIBE-EnDKER [17]	*0*	$O\left(N-r\right)$
PQS-LRIBE-DKER	*0*	$O\left(1\right)$

## Data Availability

The original contributions presented in this study are included in the article. Further inquiries can be directed to the corresponding authors.

## Abbreviations

The following abbreviations are used in this manuscript:

IBE	Identity-Based Encryption
RIBE	Revocable Identity-Based Encryption
DKER	Decryption Key Exposure Resistance
ISIS	Inhomogeneous Small Integer Solution
KGC	Key Generation Center
PQS-LRIBE-DKER	Post-Quantum Secure Lightweight RIBE scheme with DKER
LWE	Learning with Errors
